# Placebo Manipulations Reverse Pain Potentiation by Unpleasant Affective Stimuli

**DOI:** 10.3389/fpsyt.2019.00663

**Published:** 2019-09-24

**Authors:** Philipp Reicherts, Paul Pauli, Camilla Mösler, Matthias J. Wieser

**Affiliations:** ^1^Department of Psychology, University of Würzburg, Würzburg, Germany; ^2^Center of Mental Health, University of Würzburg, Würzburg, Germany; ^3^Department of Psychology, Education, and Child Studies, University of Rotterdam, Rotterdam, Netherlands

**Keywords:** placebo and nocebo effects, emotion processing, psychological pain modulation, late positive potential, somatosensory evoked potential

## Abstract

According to the motivational priming hypothesis, unpleasant stimuli activate the motivational defense system, which in turn promotes congruent affective states such as negative emotions and pain. The question arises to what degree this bottom–up impact of emotions on pain is susceptible to a manipulation of top–down-driven expectations. To this end, we investigated whether verbal instructions implying pain potentiation vs. reduction (placebo or nocebo expectations)—later on confirmed by corresponding experiences (placebo or nocebo conditioning)—might alter behavioral and neurophysiological correlates of pain modulation by unpleasant pictures. We compared two groups, which underwent three experimental phases: first, participants were either instructed that watching unpleasant affective pictures would increase pain (nocebo group) or that watching unpleasant pictures would decrease pain (placebo group) relative to neutral pictures. During the following placebo/nocebo-conditioning phase, pictures were presented together with electrical pain stimuli of different intensities, reinforcing the instructions. In the subsequent test phase, all pictures were presented again combined with identical pain stimuli. Electroencephalogram was recorded in order to analyze neurophysiological responses of pain (somatosensory evoked potential) and picture processing [visually evoked late positive potential (LPP)], in addition to pain ratings. In the test phase, ratings of pain stimuli administered while watching unpleasant relative to neutral pictures were significantly higher in the nocebo group, thus confirming the motivational priming effect for pain perception. In the placebo group, this effect was reversed such that unpleasant compared with neutral pictures led to significantly lower pain ratings. Similarly, somatosensory evoked potentials were decreased during unpleasant compared with neutral pictures, in the placebo group only. LPPs of the placebo group failed to discriminate between unpleasant and neutral pictures, while the LPPs of the nocebo group showed a clear differentiation. We conclude that the placebo manipulation already affected the processing of the emotional stimuli and, in consequence, the processing of the pain stimuli. In summary, the study revealed that the modulation of pain by emotions, albeit a reliable and well-established finding, is further tuned by reinforced expectations—known to induce placebo/nocebo effects—which should be addressed in future research and considered in clinical applications.

## Introduction

The processing of pain is prone to a variety of psychological variables, such as the affective state of an individual [for an overview, see Ref. (1)]. In this vein, it was demonstrated that emotions, induced for instance by a threat manipulation (2) or by emotionally relevant stimuli, modulate pain processing (3–6). In an earlier study, Kenntner-Mabiala and colleagues presented affective pictures for about 6 s to participants while they applied brief painful electric stimuli and registered pain ratings plus the somatosensory evoked potential (SEP) (7, 8). Results suggest that emotions modulate early pain processing as unpleasant pictures resulted in increased pain ratings and increased amplitudes of the early N1 component of the SEP relative to positive pictures. Other studies indicate that expectations regarding the characteristics of an upcoming pain stimulus also determine the processing of nociceptive stimulation and the resulting pain perception (9, 10). The same is true for placebo and nocebo effects on pain; however, here, expectations focus on the *effect of an intervention*, which is expected to decrease (placebo) or increase pain (nocebo) (10–12). Expectations causing placebo and nocebo effects can be induced by verbal instructions suggesting a pain-modulating effect and/or by the actual experience of pain relief or pain exacerbation (placebo/nocebo conditioning) associated with a certain treatment or—experimental—intervention (13–17).

In a recent study, we investigated the respective contribution of expectations and prior experiences on the formation of placebo effects. To this end, we introduced a new, completely psychological placebo manipulation, which ensured that participants had not encountered the placebo agent before and thus had no *a priori* expectation. We employed a common approach in placebo and nocebo research that is a placebo/nocebo instruction followed by a reinforcing conditioning phase, during which placebos were combined with lower and nocebos with stronger pain stimuli. Three experimental conditions were compared: Participants were either only informed of an analgesic/pro-algesic effect they were about to encounter, or participants actually experienced different levels of pain in a conditioning procedure, or participants received both, an instruction informing about a pain-modulating effect, which received support during a subsequent conditioning phase. We found that the latter condition, i.e., expectation plus concordant conditioning, was capable in modifying subjective and physiological indices of pain, even though the placebo/nocebo manipulation was lacking pharmacological plausibility, since we instructed participants that “watching certain black and white stripe patterns were found to have a pain augmenting/easing effect,” respectively (18). These findings corroborate the critical role of higher-order cognitions for the modulation of pain.

Placebo and nocebo effects, however, are by no means restricted to pain. Significant modifications have been found for various somatic symptoms (12) and also for the perception of emotions. For example, Petrovic and colleagues found that subjective and neuronal responses to unpleasant affective pictures were reduced if participants believed they had received an anxiolytic medication (19). Based on the involved brain areas, the authors assume similar underlying mechanisms in placebo effects altering emotion and pain alike. More recently, Schienle and colleagues (20) demonstrated reduced feelings of disgust paralleled by reduced insular activation when participants thought they took a herbal drug against disgust symptoms. In a related manner, findings from research on reversal learning show placebo- and nocebo-like effects on emotion processing. For instance, threat responses following the presentation of previously established conditioned threat cues (CS+), which were paired with aversive electrical stimuli, are reduced, if participants receive a verbal instruction that the cue is no longer indicative of danger (21). Similarly, although the presentation of emotional facial expressions reliably evokes positive or negative affective responses in an observer, verbal instruction about potential danger being indicated by a certain face category leads to defensive responding irrespective of face valence (e.g., happy or fearful faces announcing an aversive outcome) (12). These results nicely demonstrate that emotional responses can be shaped *top–down* by cognitive representations of superordinate functions.

Interestingly, placebo and nocebo effects often come along with emotional responses, such as anticipatory anxiety (nocebo) or positive feelings of relief and reward (placebo), which—to some degree—might mediate the modulation of (pain) symptoms (22–24). For instance, Aslaksen and colleagues showed that a nocebo instruction suggesting hyperalgesic effects caused by an applied cream led to a pain increase, which was meditated by subjective and physiological indices of stress (25). However, when applying a mere conditioning procedure without explicit placebo or nocebo instructions, the role of negative affect might be less relevant (26). Just recently, Geers and colleagues found that the experimental induction of positive mood by watching a pleasant movie clip was capable to block a pain increase by a verbal nocebo suggestion (27). Despite all these findings, so far, little research explored the interaction of emotions on the one hand and placebo/nocebo manipulations on the other when modulating pain.

In the present study, we aimed at investigating whether the genuine pain-modulating effect of unpleasant affective pictures is sensitive to a placebo or nocebo manipulation. To this end, we compared two groups of participants who received either a placebo or nocebo manipulation related to unpleasant pictures. The nocebo group was instructed that watching unpleasant pictures leads to an increased perception of pain in line with findings from the literature (nocebo instruction), and during a later conditioning procedure, they actually experienced relatively more intense pain stimuli when watching the “nocebo” pictures. The placebo group was told the exact opposite, namely, that unpleasant pictures cause a decreased perception of pain. Thereafter, participants experienced relatively less intense pain stimuli when watching the “placebo” pictures. In addition to pain reports, we measured the electroencephalogram (EEG) that allowed us to analyze neurophysiological correlates of pain perception (N1 and P2 component of the SEP as mentioned earlier) and processing of the emotional pictures by means of visually evoked potentials (28). One component of the visually evoked potential following the presentation of emotional relevant stimuli is the late positive potential (LPP)—a positive signal deflection most prominent at centro-parietal electrode sites—which was found to be a sensitive measure for emotional intensity (arousal) of presented pictures (29–31).

We hypothesized that unpleasant picture stimuli generally increase pain processing; however, this effect is modulated by reinforced expectations induced by a placebo/nocebo manipulation. Specifically, we expect that a placebo manipulation (verbal instruction + placebo conditioning) reduces or even reverses the pain-augmenting effect of unpleasant pictures. This might lead to lower pain ratings and SEPs for unpleasant compared with neutral pictures. Further, the placebo manipulation might become evident also in altered neurophysiological correlates of unpleasant affective pictures processing, namely, by a lack of LPP modulation or even higher amplitudes for neutral compared with unpleasant pictures.

## Materials and Methods

### Sample

Forty-two participants were recruited from the University of Würzburg and received course credit or €20 as compensation. Two participants needed to be excluded due technical problems during data acquisition, leaving 40 participants in the final analysis, 20 participants in the nocebo group (10 females) and 20 participants in the placebo group (10 females). All subjects had normal or corrected-to-normal vision, reported no current or prior history of chronic pain, neurological or psychiatric disorders (self-report), and did not take any analgesic medication prior to the experiment. Participants first read detailed instructions about the experiment and signed the informed consent before taking part in the experiment. Participants filled out questionnaires on current positive and negative affect (Positive Affect/Negative Affect Schedule) (32), on state and trait anxiety (State/Trait Anxiety Inventory) (33), on pain catastrophizing (Pain Catastrophizing Scale) (34), on sensitivity for pain (35), on dispositional optimism and pessimism (Life Orientation Test—Revised) (36), and on anxiety of pain related symptoms (Pain Anxiety Symptom Scale) (37). Questionnaire scores of both groups were similar except for state anxiety, which was higher in the placebo group (see **Table 1**). All procedures were approved by the institutional review board of the medical faculty of the University of Würzburg.

**Table 1:** Sample Characteristics.

Measure	Nocebo (n = 20)		Placebo (n = 20)		*F*	*p*	
	m	sd	m	Sd			
**Pain Stimulation in mA**	2.79	1.01	2.91	1.74	.07	.79	
**Pain Stimulus Rating (0–10)**	6.25	0.97	6.45	1.19	.34	.56	
**PANAS Positive**	35.50	21.50	32.00	5.46	.50	.48	
**PANAS Negative**	13.95	4.17	12.70	2.36	1.36	.25	
**STAI State**	38.65	5.81	34.30	4.61	6.88	.01	*
**STAI Trait**	39.20	7.87	35.50	5.99	2.80	.10	
**LOT**	8.45	3.47	7.45	2.48	1.10	.30	
**PSQ**	4.01	1.11	3.96	1.43	.01	.91	
**PCS**	20.80	7.02	17.15	7.16	2.65	.11	
**PASS-D**							
**Age**	23.50	2.48	24.25	2.26	1.01	.32	
**Post Experimental Survey asking for the:**							
**…Effect of Unpleasant Pictures on Pain (-4 to +4)**	1.90	1.59	-1.90	0.91		< .001	**
**…Effect of Neutral Pictures on Pain (-4 to +4)**	-0.35	0.75	0.90	1.25		< .001	**

Both groups consisted of 10 women and men; PANAS, Positive Affect/Negative Affect Schedule; STAI, State/Trait Anxiety Inventory; LOT, Life Orientation Test; PSQ, Pain Sensitivity Questionnaire; PCS, Pain Catastrophizing Scale; PASS-D, Pain Anxiety Symptom Scale; *p < .05; **significant Mann–Whitney U Test.

### Visual Stimuli

Participants watched 40 emotional pictures (twice), which were drawn from the International Affective Pictures System (38), comprising 20 neutral (International Affective Pictures System catalog numbers: 2095, 3170, 3180, 3230, 3261, 3500, 3530, 6212, 6256, 9040, 9050, 9163, 9250, 9300, 9321, 9413, 9419, 9901, 9921, and 9925) and 20 unpleasant pictures (2038, 2191, 2383, 2393, 2396, 2514, 2595, 2749, 2850, 2870, 2880, 5390, 5731, 5870, 7002, 7100, 7130, 7493, 7550, and 7590). Pictures were presented for 6 s interleaved by a central fixation cross present for 2–3 s (randomized). Picture order was randomized with the restriction of no more than two consecutive pictures of the same valence. Visual stimuli were projected centrally on a screen of 2 × 3.22 m (Powerwall), at 2.0-m distance from the participant’s chair.

### Electrical Pain Stimulation

Electrical pain stimuli were delivered on the left calf of the participants *via* a surface bar electrode with two stainless steel disk electrodes (8-mm diameter, 30-mm spacing), using a constant-current stimulator (Digitimer DS7A, Digitimer Ltd., Welwyn Garden City, UK). The intensity of the electrical stimulus was adjusted to the participants’ individual pain threshold. During thresholding, participants were asked to rate electrical stimuli of two ascending and two descending series starting from 0 mA applying steps of ±0.5 mA, respectively, on a 11-point scale ranging from 0 (no pain at all) to 10 (unbearable pain). Stimulus intensities rated with a 4 (just noticeable pain) were averaged, and 1 mA was added to the final stimulus intensity to reassure a moderate pain level. The final stimulation intensity was again rated on a 10-point scale (see **Table 1**). During the experiment, two different stimulation intensities were used, which varied with regard to the number of consecutive single pulses (train length). Low intense stimuli consisted of three square pulses (pulse length 2 ms) and an inter-pulse interval of 4 ms, high intense stimuli instead consisted of 10 square pulses. During the test phase and the threshold procedure, high intense stimuli were delivered, and during the conditioning phase, both low and high intense stimuli were used; see the procedure section for further details.

### Electroencephalogram Recording and Evoked Potentials

Electrophysiological data were recorded from 32 active electrodes (ActiCap; Brain Products, Munich, Germany) with a sampling rate of 1,000 Hz, placed according to the international 10–20 system (C3, C4, CP1, CP2, CP5, CP6, Cz, F3, F4, F7, F8, FC1, FC2, FC5, FC6, Fp1, Fp2, Fz, O1, O2, Oz, P3, P4, P7, P8, Pz, T7, T8, TP10). FCz was used as online reference, and data were off-line re-referenced to an average reference. Vertical (above and below the left eye) and horizontal (at the outer canthi of both eyes) electrooculogram was recorded. Electrode impedance was kept below 5 k Ohm, and the online band-pass filter was set to 0.01 to 250 Hz. Data were collected using a Brain-Amp-MR amplifier (Brain Products) and the software Brain Vision Recorder Version 1.05 together with ActiCap Control Software (Brain Products). Off-line EEG analysis was performed using Brain Vision Analyzer Version 2.1 (Brain Products). EEG was filtered (0.1–30 Hz) and corrected for horizontal and vertical ocular artifacts (39). Trials exceeding a transition threshold of 50 µV (sample to sample) or an amplitude criterion of ±100 µV were excluded from further analysis. For the analysis of the picture evoked LPP, epochs registered 100 ms before to 2,000 ms after picture onset were extracted and baseline corrected with reference to the mean baseline interval (100 ms before picture onset). The LPP was scored at the parietal electrode Pz and quantified as mean activity from 700- to 1,000-ms post picture onset, according to visual inspection of the scalp topographies and the literature (28, 31). For the analysis of the SEP following electrical stimulation, epochs registered 100 ms before to 1,000 ms after electrical stimulation (first pulse) were extracted, baseline-corrected, and averaged analog to the procedure of the LPP. Two components of the SEP were analyzed, that is, the N1 and P2, which were scored as mean activity at the Cz electrode in a time window from 75 to 125 ms and 200 to 330 ms, respectively (3, 8). For statistical analysis, all event-related potential components were averaged per participant across all artifact-free picture and pain epochs of the conditioning and test phase, respectively.

### Pain Ratings

After each electrical stimulation, pain intensity and unpleasantness ratings were obtained using a digital visual analog scale. Ratings were converted off-line to values between 0 and 100. The scale for pain intensity ratings was labeled “not painful at all” at the left end and “extremely painful” at the right end of the scale, and for pain unpleasantness, the scale ranged from “not unpleasant at all” to “extremely unpleasant.”

### Procedure

**Figure 1:**
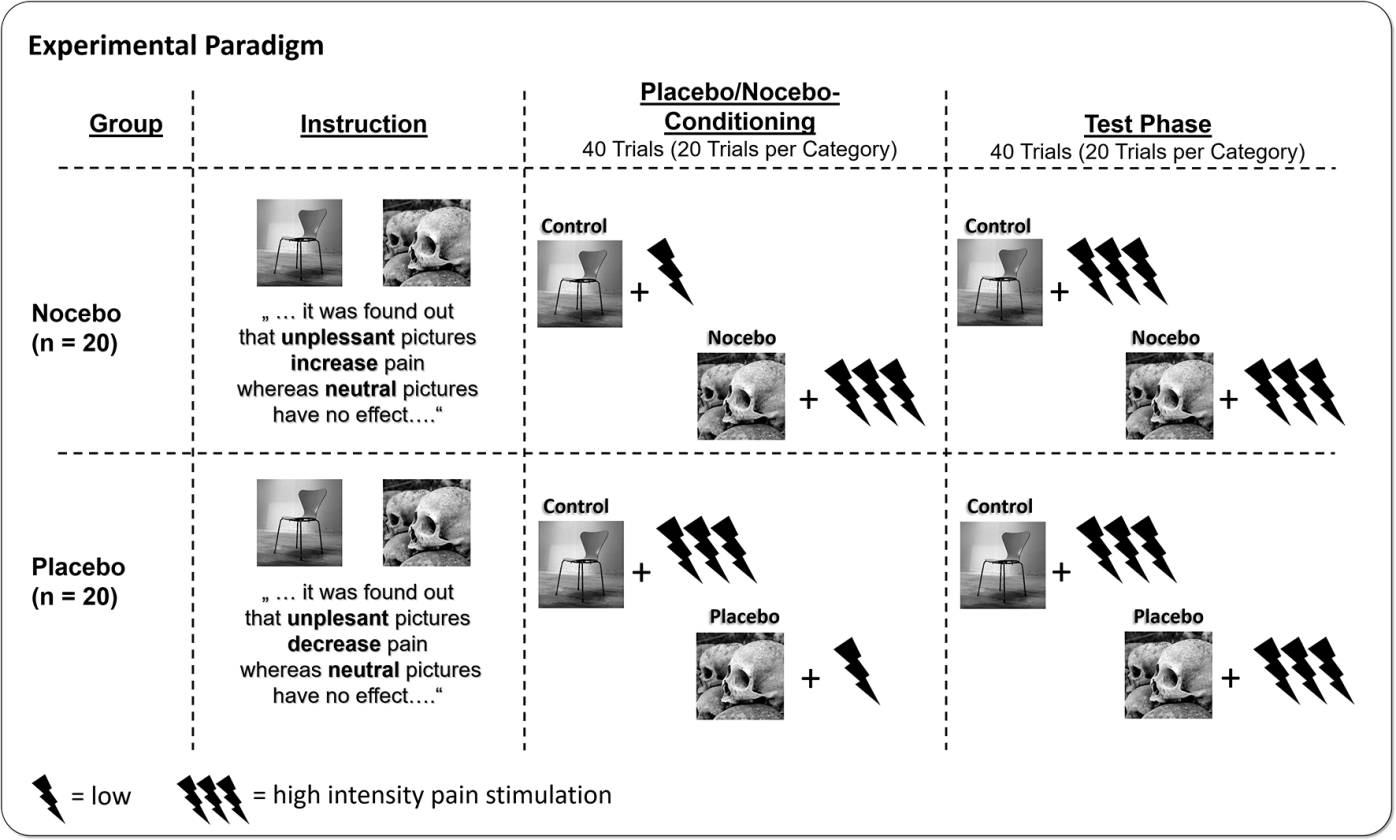
Participants were either instructed that watching unpleasant affective pictures would increase pain (nocebo group), or the exact opposite, that watching unpleasant pictures would decrease pain (placebo group) relative to neutral pictures. Afterwards, participants underwent placebo/nocebo conditioning, where unpleasant and neutral pictures were paired with either high- or low-intensity electrical pain stimuli, in line with the placebo or nocebo instruction provided previously. In the following test phase, participants watched the placebo or nocebo and neutral pictures again but received always high-intensity pain stimuli.

After arrival, participants were assigned to one of the two experimental groups (nocebo vs. placebo)—taking into account the participants’ gender—following an *a priori* randomization performed by the experimenter. According to the respective experimental condition, participants were instructed that during the experiment, they would watch a series of unpleasant and neutral pictures, which—in line with recent findings in the literature—very likely would change their perception of concurrently administered painful electrical stimuli. The nocebo group was told that unpleasant pictures would *increase* the perception of pain, while neutral pictures had no influence on pain at all. The placebo group instead was told that unpleasant pictures would result in a *decreased* perception of pain compared with neutral pictures, which would leave the perception of pain unchanged. Participants were seated 2.0 m in front of the screen and started the experiment. Unbeknownst to them, the experiment consisted of two parts, the conditioning phase, which was followed without interruption by the test phase. During conditioning, participants of the nocebo group watched neutral pictures and received the low-intensity pain stimuli and unpleasant pictures paired with high-intensity pain stimuli. This association was reversed for participants of the placebo group; here, participants were administered the low-intensity pain stimuli during unpleasant and the high-intensity stimuli during neutral picture presentation. Following the logic of previous placebo manipulation, this procedure should reassure the participants that the instruction they were given in the beginning of the experiment actually hold true and pain perception was modulated accordingly. During the test phase, participants of both groups always received the same, high-intensity pain stimulation, combined with neutral and unpleasant pictures (see **Figure 1**). After each trial, participants rated the electrical stimulus for pain intensity and unpleasantness. In total, participants completed 80 trials, which is 20 repetitions of unpleasant and neutral pictures per phase. In the end, participants filled out a post experimental survey asking how they evaluate the effect of unpleasant and neutral pictures on pain using a 9-point scale ranging from +4 (very pain increasing) to 0 (no effect on pain) to -4 (very pain reducing). Stimulus presentation was controlled by the software Presentation (Neurobehavioral Systems, Albany, CA, USA).

### Statistical Analysis

Pain ratings (pain unpleasantness, pain intensity) and amplitudes of the SEP components (N1 and P2) were analyzed separately for the conditioning and the test phase. During the conditioning phase, a 2 × 2 repeated measures analysis of variance (ANOVA) including the within-subjects factors *Stimulation Level* (high vs. low intensity stimulation, irrespective of picture category) and the between-subjects factor *Group* (nocebo vs. placebo) was applied. During the test phase, pain responses following identical stimulation intensities were analyzed using the within- subjects factor *Emotion* (unpleasant vs. neutral pictures) and the between-subjects factor *Group*. LPPs were analyzed using a 2 × 2 × 2 repeated measures ANOVA including the within-subjects factor *Emotion* (unpleasant vs. neutral pictures), *Phase* (conditioning vs. test phase) to capture potential changes across the time course of the experiment, and the between-subjects factor *Group*. Significant interaction was explored using follow-up ANOVAs. The significance level was set to .05 (two-tailed); for follow-up ANOVAs, a corrected alpha of *p* < .025 was considered. As a measure of effect size, we report partial *η*². Normal distribution of the analyzed data can be assumed for 93% of the variables (Shapiro–Wilk’s tests), due to the robustness of the repeated measures ANOVA against violations of data normality (40); its usage seems appropriate in the present case.

## Results

### Pain Ratings—Conditioning Phase

**Figure 2:**
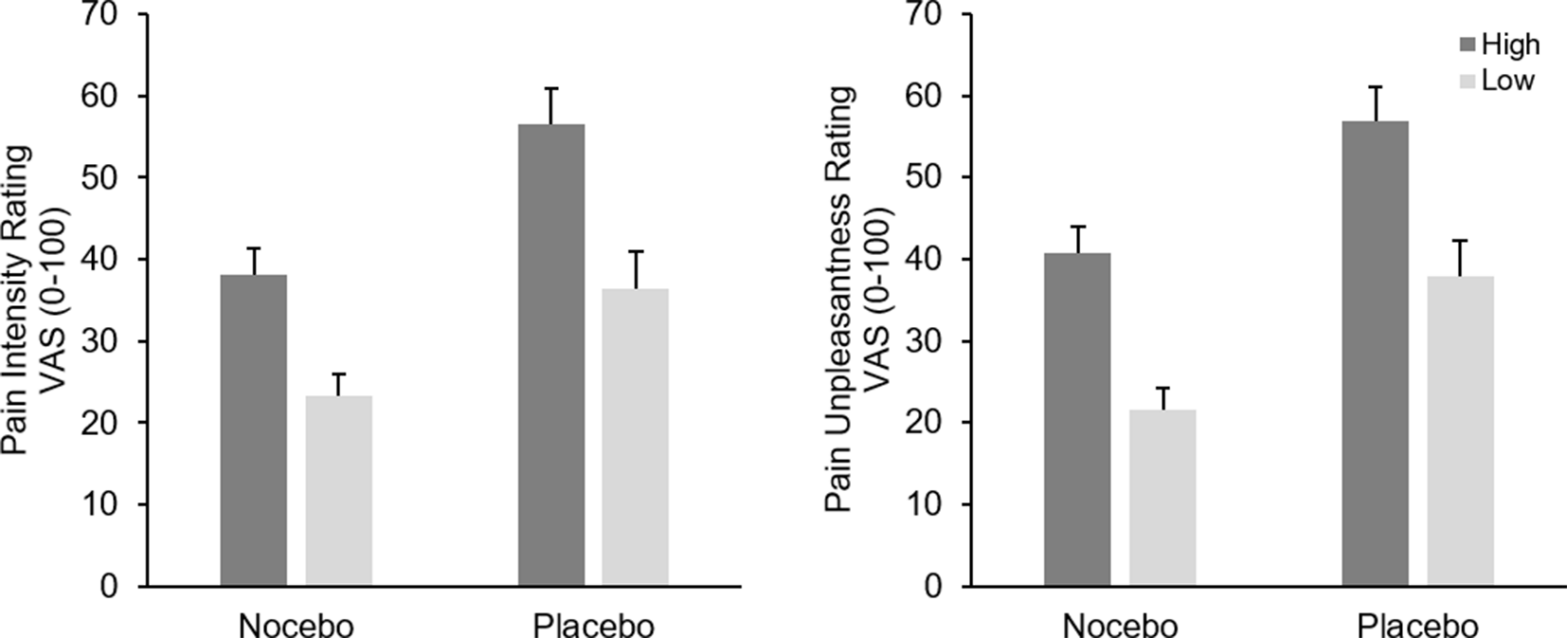
Mean pain intensity (left) and unpleasantness (right) ratings (+SEM) in the conditioning phase separately for stimulus intensity (high vs. low) and experimental group (nocebo vs. placebo). All within group comparisons and the between factor were significant (*p* < .05).

Analysis of pain intensity ratings revealed a significant main effect of *Stimulation Level* *F*(1, 38) = 152.54, *p* < .001, *η* *_p_*² = .80, as a result of higher pain ratings following more intense electrical stimulation. The interaction of *Stimulation Level* × *Group* was only marginally significant, *F*(1, 38) = 3.49, *p* = .07, *η* *_p_*² = .08, presumably indicating a more pronounced differentiation between the two stimulation intensity for the placebo group. The factor *Group* was significant, *F*(1, 38) = 9.36, *p* = .004, *η* *_p_*² = .20, due to higher pain ratings in the placebo compared with the nocebo group (*M* = 46.54 vs. *M* = 30.64), see **Figure 2**.

Analysis of pain unpleasantness ratings returned a similar picture, participants clearly differentiated between the two different pain stimuli as indicated by the significant main effect of *Stimulation Level*, *F*(1, 38) = 113.96, *p* < .001, *η* *_p_*² = .75, however the interaction of *Group* x *Stimulation* was not significant, *F*(1, 38) = 0.01, *p* = .99, *η* *_p_*² < .01. Again, participants in the placebo group reported higher pain in general, *F*(1, 38) = 10.72, *p* = .002, *η* *_p_*² = .22, (*M* = 47.32 vs. *M* = 31.12), see **Figure 2**.

### Pain Ratings—Test Phase

**Figure 3:**
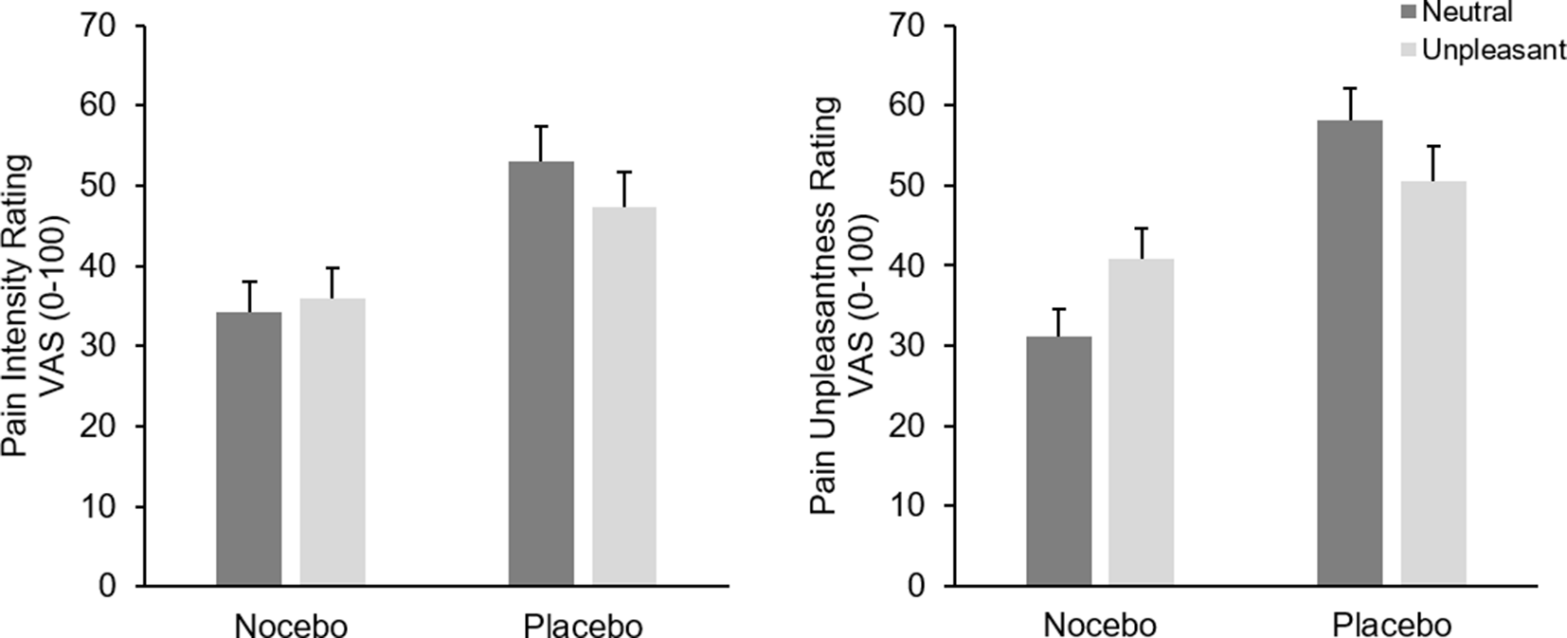
Mean pain intensity (left) and unpleasantness (right) ratings (+SEM) in the test phase separately for picture category (neutral vs. unpleasant) and experimental group. All within group comparisons—except for pain intensity ratings of the nocebo group—and the between factor were significant (*p* < .05).

Analysis of pain intensity ratings revealed a marginal significant main effect of *Emotion* *F*(1, 38) = 3.66, *p* = .06, *η* *_p_*² = .09, which was further qualified by a significant interaction of *Emotion* × *Group*, *F*(1, 38) = 11.72, *p* < .001, *η* *_p_*² = .24. Participants in the placebo group rated pain during neutral pictures significantly higher than during unpleasant pictures, *F*(1, 19) = 11.97, *p* = .003, *η* *_p_*² = .39, while the same comparison failed significance in the nocebo group, *F*(1, 19) = 1.41, *p* = .25, *η* *_p_*² = .07. The factor *Group* was also significant, *F*(1, 38) = 6.94, *p* = .01, *η* *_p_*² = .15, resulting from generally higher pain ratings in the placebo group (*M* = 50.17 vs. *M* = 35.07), see **Figure 3**.

Analysis of pain unpleasantness ratings showed no main effect of *Emotion F*(1, 38) = 0.46, *p* = .50, *η* *_p_*² = .01; however, the interaction of *Emotion* × *Group* was significant, *F*(1, 38) = 31.67, *p* < .001, *η* *_p_*² = .45. Separate ANOVAs for each group revealed a significant main effect of *Emotion* for both the nocebo *F*(1, 19) = 22.10, *p* < .001, *η* *_p_*² = .54 and the placebo groups, *F*(1, 19) = 11.13, *p* = .003, *η* *_p_*² = .37. However, while participants in the nocebo group rated pain stimuli higher during unpleasant compared with neutral pictures (*M* = 40.92, vs. *M* = 31.17), participants in the placebo group showed the exact opposite pattern, namely, higher pain unpleasantness ratings while seeing neutral (*M* = 58.15) compared with unpleasant pictures (*M* = 50.50). Again, the placebo group showed generally higher pain unpleasantness ratings compared to the nocebo group, *F*(1, 38) = 11.89, *p* = .001, *η* *_p_*² = .24, (*M* = 54.33 vs. *M* = 36.45), see **Figure 3**.

### Somatosensory Evoked Potentials—Conditioning Phase

**Figure 4:**
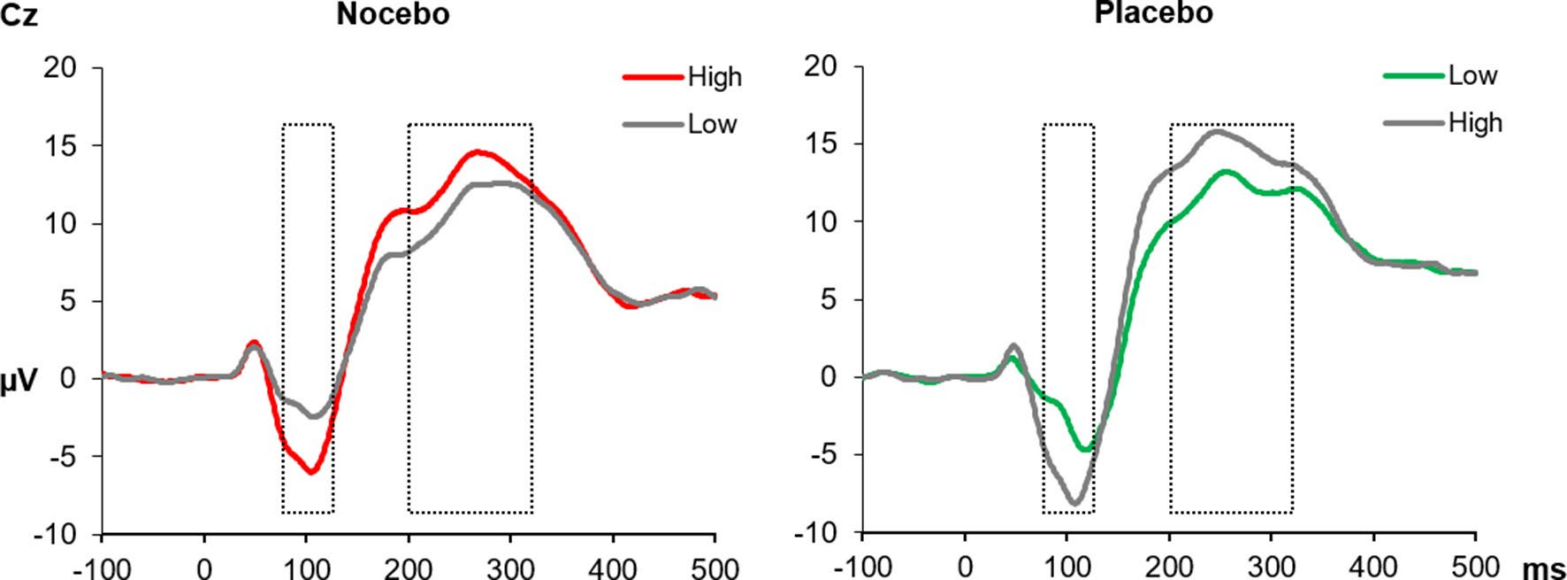
The SEPs at the Cz electrode elicited by electrical pain stimuli during the conditioning phase. In the nocebo group (left), unpleasant pictures (red line) were paired with high-intensity pain stimuli, and in the placebo group, unpleasant pictures (green line) were paired with low-intensity pain stimuli. The gray lines represent neutral pictures, combined with either high- or low-intensity stimuli. The N1 (mean activity 75–125 ms) and the P2 (200–330 ms) components were significantly increased for high- compared with low-intensity pain stimuli in both experimental groups. All within group comparisons *p* < .05.

As expected, during the conditioning phase, the physically more intense pain stimuli resulted in elevated SEP amplitudes. This was true for the early N1 component, *F*(1, 38) = 73.14, *p* < .001, *η* *_p_*² = .66, and the subsequent P2, *F*(1, 38) = 19.11, *p* < .001, *η* *_p_*² = .34. For both components, neither the interaction [**N1**, *F*(1, 38) = 0.70, *p* = .41, *η* *_p_*² = .02; **P2**, *F*(1, 38) = 0.91, *p* = .35, *η* *_p_*² = .02] nor the factor *Group* reached significance [**N1**, *F*(1, 38) = 1.14, *p* = .29, *η* *_p_*² = .03; **P2**, *F*(1, 38) = 0.43, *p* = .52, *η* *_p_*² = .01], see **Figure 4**.

### Somatosensory Evoked Potentials—Test Phase

**Figure 5:**
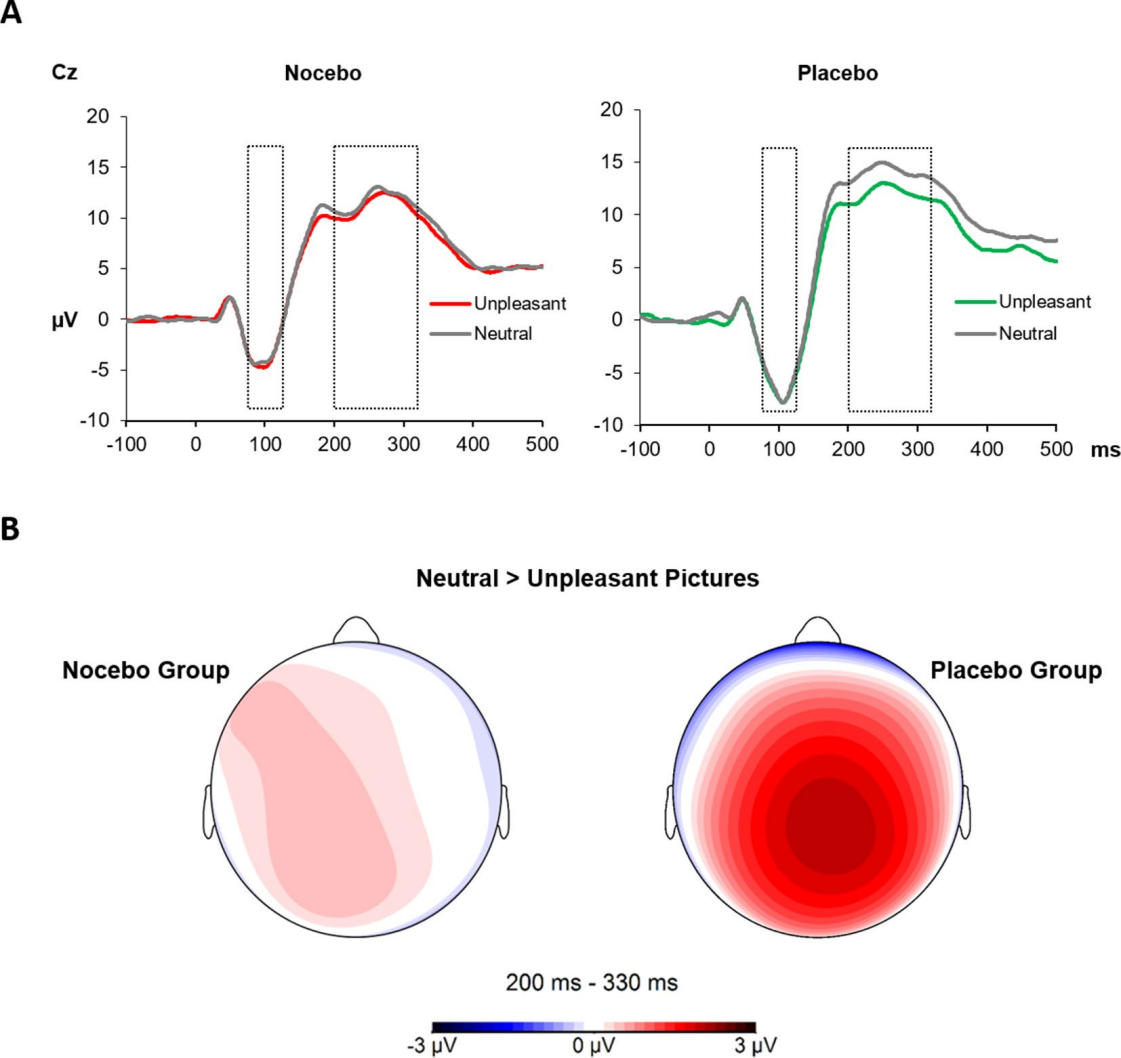
**(A)** The SEPs at the Cz electrode elicited by electrical pain stimuli during the test phase. The N1 (75–125 ms) component showed no modulation by picture category or across groups. The P2 (200–330 ms) component instead was significantly decreased for electrical stimuli paired with unpleasant pictures in the placebo group only. **(B)** Scalp topography 220–300 ms for the difference of neutral and unpleasant pictures, separately for each group.

Analysis of **N1** amplitudes during the test phase—when pain stimuli had always the same intensity—revealed neither a significant main effect of *Emotion F*(1, 38) = 0.01, *p* = .93, *η* *_p_*² < .01 nor a significant interaction *F*(1, 38) = 0.47, *p* = .50, η*_p_*² = .01. The between factor was marginally significant, *F*(1, 38) = 3.27, *p* = .08, *η* *_p_*² = .08, likely due to more pronounced amplitudes in the placebo (*M* = -6.33) compared with nocebo group (*M* = -3.71). The **P2** component similarly revealed no significant effect of *Emotion F*(1, 38) = 2.75, *p* = .11, *η* *_p_*² < .07; however, the interaction of *Group* × *Emotion* was significant, *F*(1, 38) = 7.44, *p* = .01, *η* *_p_*² < .16. Separate ANOVAs for each group showed a significant main effect of *Emotion* only for the placebo group, *F*(1, 19) = 6.64, *p* = .02, *η* *_p_*² = .26, due to higher mean amplitudes following neutral (*M* = 14.02) compared with unpleasant pictures (*M* = 11.89). The same analysis returned a nonsignificant main effect of *Emotion F*(1, 19) = 1.04, *p* = .32, *η* *_p_*² = .05 for the nocebo group¸ see **Figure 5**.

### Visually Evoked Potentials During Conditioning and Test Phases

**Figure 6:**
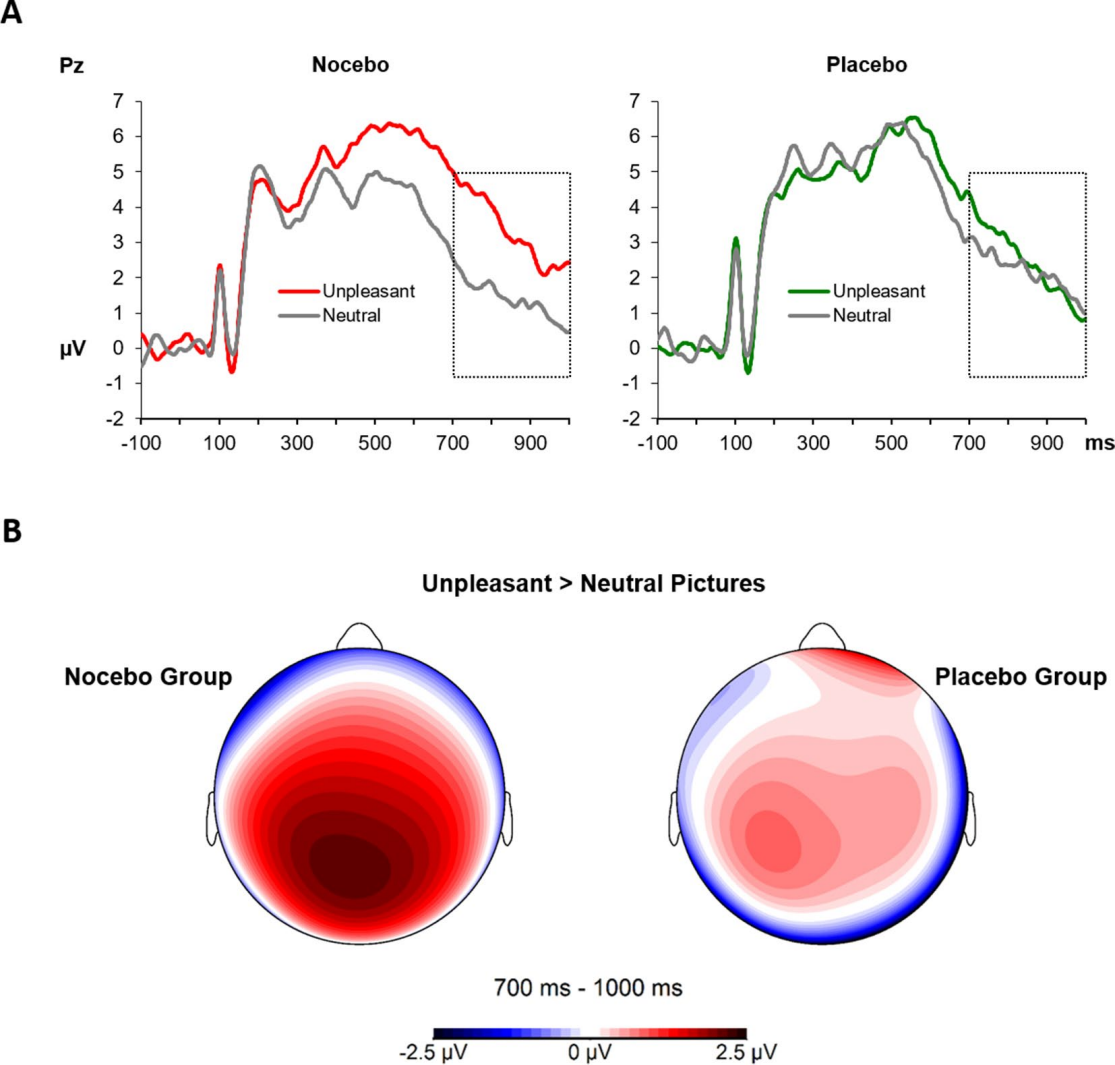
**(A)** The LPPs at the Pz electrode averaged across conditioning and test phases, separately for neutral and unpleasant pictures and split by experimental group. The LPP between 700 and 1,000 ms (marked time window) was higher for unpleasant compared with neutral pictures in the nocebo group only (*p* = .002). **(B)** Scalp topography 700–1,000 ms for the difference of unpleasant and neutral pictures, separately for each group.

The 2 × 2 × 2 ANOVA for the analysis of the visually evoked LPPs revealed a significant main effect *Phase F*(1, 38) = 15.50, *p* = .001, *η* *_p_*² = .29, which was the result of higher LPP amplitudes during the test compared with the conditioning phase (*M* = 1.68 vs. *M* = 3.00). Furthermore, the significant main effect of *Emotion* *F*(1, 38) - 6.79, *p* = .01, *η* *_p_*² = .15, was further qualified by a close to significant two-way interaction of *Emotion* × *Group*, *F*(1, 38) - 3.95, *p* = .054, *η* *_p_*² = .09. Follow-up ANOVAs separately for each group revealed a significant main effect of *Emotion F*(1, 19) - 13.39, *p* = .002, *η* *_p_*² = .41 for the nocebo group, which is the result of elevated LPP amplitudes for unpleasant compared with neutral pictures. Interestingly, for the placebo group, the factor *Emotion* was far from being significant, *F*(1, 19) - 0.16, *p* = .70, *η* *_p_*² < .01, see **Figure 6**. No other main effect or interaction reached significance, all *p*s > .22.

## Discussion

In the current study, we addressed the question whether a placebo/nocebo manipulation does alter the pain-enhancing effect of emotions elicited by unpleasant picture stimuli, and if so, neurophysiological correlates of emotions processing were changed accordingly. Results demonstrate lower pain ratings for unpleasant pictures introduced as placebo compared with neutral control pictures. Further, in the placebo group only, negative pictures led to reduced P2 amplitudes of the SEP. In the nocebo group, in line with classical findings, unpleasant pictures led to more pronounced LPP amplitudes than neutral pictures. In the placebo group instead, pleasant (placebo) and neutral (control) pictures led to similar neurophysiological responses, suggesting that the placebo manipulation already affected the processing of the emotional stimuli and, consequently, processing of the pain stimuli.

### Pain Modulation by Pictures Indicating Placebo Hypoalgesia or Nocebo Hyperalgesia

Pain ratings and neurophysiological pain responses during the conditioning phase of the experiment demonstrated a clear differentiation between the two stimulus intensities, which suggests a successful manipulation of the participants’ actual experience in line with the idea of reinforced expectations often used in placebo/nocebo designs (16, 18, 41). The high-intensity stimuli were rated as more painful and unpleasant and evoked larger amplitudes of the N1 and P2 components of the SEP in both groups. Regarding the pain intensity ratings, the difference between neutral and unpleasant pictures tended to be even stronger in the placebo group, which is a first hint for a critical role of top–down-driven expectations rather than invariant effects of emotions on pain: following the concept of motivational priming (5, 42), one might have expected the pain-increasing effect of unpleasant pictures to be *enhanced* in the nocebo group. Instead, the placebo manipulation led to an even more pronounced differentiation, suggesting a more prominent role of the reinforced expectations than of the unpleasant pictures. The generally elevated pain ratings in the placebo group might to some degree be also the consequence of the experimental manipulation, but see the limitation section for further discussion.

The results from the test phase demonstrate even more clearly the interplay of our placebo/nocebo manipulation and the modulation of pain by emotions. Participants of the nocebo group demonstrated the well-known pain-augmenting effect of unpleasant pictures (43). The placebo group, however, reveals a completely reversed pattern. Here, the unpleasant pictures, introduced as having a pain-easing effect, led to significantly *reduced* pain intensity and unpleasantness ratings—of physically identical pain stimuli—compared with the neutral pictures. This indicates that the placebo expectation in combination with the conditioning procedure was able to reverse the pain-increasing effect of negative emotions, reflecting an efficient top–down control of pain processing.

There is a long-standing debate regarding the role of expectancy and learning, i.e., conditioning underlying the formation of placebo effects [see for instance (44, 16)], and some even question whether placebo effects per se are anything but conditioning effects and suggest to drop the concept in general (45). With regard to our present results, it is hard to tell whether the instruction in the beginning of the experiment or the placebo acquisition phase contributed to greater extent to the final placebo effect. A previous study by our group showed that in case of so to say psychologically mediated placebo/nocebo agents, both aspects are crucial. It might be interesting to test whether the present findings would replicate if only a conditioning procedure or placebo instruction was applied. Although research on expectancy effects on psychological pain modulation is rather sparse, the effect of placebo and nocebo expectations was repeatedly shown for pharmacological pain interventions. For instance, it was demonstrated that the same dosage of pain medication is more effective if it is administered in a so-called open fashion—that is, a patient is well aware of receiving a medication, which generates a robust expectation for analgesia—in contrast to a hidden application without explicit knowledge of the patient (46). On the contrary, a nocebo expectation is capable to abolish the effectiveness of a highly potent analgesic medication (47). Accordingly, the present findings suggest that the same might be true if the pain-modifying mechanism at question is based on psychological processes, here emotion-based pain modulation.

### Placebo Expectations and Emotion Processing

Analysis of the LPPs, elicited by the picture stimuli, showed that participants in the nocebo group clearly differentiated between neutral and unpleasant pictures in line with previous studies on neurophysiological correlates of affective picture processing, which demonstrated a preferential processing of threatening stimuli (30, 31, 48, 49). However, the placebo group failed to exhibit discriminative LPPs for neutral versus unpleasant pictures, which might be due to an integration of emotional picture content and their alleged effect on pain. We suppose that the placebo manipulation changed the functional representation of the unpleasant pictures, since according to the instruction, those were now indicative for a positive outcome, which likely rendered them as less threatening. In accordance with this interpretation, Bradley and colleagues found that physiological responses following the presentation of emotional pictures change if picture valence—positive vs. negative—operates as a cue for threat vs. safety, respectively (50). Threat cues, provoked stronger physiological defense reactions, irrespective of the emotional picture content. In a similar paradigm where positive and unpleasant pictures alternatingly served as threat or safety cues, analysis of the LPPs demonstrated elevated amplitudes for pictures indicating potential danger (51). Altogether, these findings suggest that affective picture processing and emotional responding is susceptible to a top–down-driven modulation of motivational/functional significance. These results are further in line with a finding from research on emotion regulation, demonstrating that changing the meaning of an emotional relevant scene for instance by applying an alternative interpretation (reappraisal) leads to altered subjective and neurophysiological responses following picture processing (30, 52, 53).

### Neurophysiological Pain Responses While Watching Pictures Indicating Placebo or Nocebo

SEPs during the test phase demonstrated no modulation of the early N1 component, neither in the nocebo group nor in the placebo group. However, previous studies found a significant modulation of the N1 solely for the comparison of unpleasant with positive pictures (3, 8). Accordingly, the contrast between neutral and unpleasant pictures was likely not strong enough, which might explain the lacking N1 modulation, especially in the nocebo group. Similarly, studies on placebo effects measuring neurophysiological responses to short laser beams found no modulation of early components of the LEP (54, 55). The P2 component instead was modulated by the picture category, but only in the placebo group, such that unpleasant compared with neutral (control) pictures led to a significantly reduced amplitude. This is in line with earlier findings demonstrating that emotional compared with neutral pictures reduce the P2 following electrical stimuli (3, 8). Furthermore, studies investigating placebo effects on LEPs found that a placebo manipulation reduced the P2 component or N2/P2 complex, respectively (54, 55). Given that participants in the placebo group showed little differentiation between the picture categories as indicated by similar LPP amplitudes, this might demonstrate an interference of emotion processing by the placebo manipulation. We conclude that the reduction of the P2 component likely is driven more strongly by a placebo effect than by the arousing content of the pictures. In the nocebo group, however, the instructed pain-augmenting effect of unpleasant pictures did not provoke any conflict between picture content (negative) and functional significance (negative). Here, in line with previous studies on nocebo-like cueing effects reporting elevated LEPs (56), the experimental manipulation probably led to an increase of the P2 component during unpleasant picture presentation, which compensated the expected P2 decrease by high-arousing pictures found previously. Yet, the nocebo effect apparently was not strong enough to produce a significant potentiation of the P2 by unpleasant *nocebo* pictures, exceeding the responses following the neutral control pictures.

### Limitations

Although the ratio of female and male subjects was equal within and across groups, due to the small total sample, a moderation of the reported findings by the participants’ gender cannot be excluded. Future studies should incorporate larger sample sizes to explore gender effects in more detail and to control for the sometimes-high variability in placebo and nocebo designs. Furthermore, even though the experimental groups varied only very little with regard to the individual pain threshold and later on administered pain stimuli, participants of the placebo group reported higher pain intensity and unpleasantness ratings, in general, despite similar SEPs amplitudes. Results of the post-experimental ratings—where participants of the placebo group indicated a relative pain-increasing effect of neutral pictures compared with the nocebo group—might be suggestive for an overall overestimation of pain in the placebo group, leading to elevated pain ratings, see **Table 1**. However, evidence for this interpretation is inconclusive and might be corroborated in future studies, obtaining measures of the participant’s expectation already in the beginning of the experiment. In a similar vein, we decided against trial-by-trial affective ratings of the emotional picture stimuli. This might have been informative with regard to the findings from the visual evoked potentials but, at the same time, led to excessive length of the whole experiment. Future studies should complement physiological affective responses by subjective measures of emotion and expand the stimulus set by a positive valence category. With regard to state affect, participants in the nocebo group presented somewhat higher anxiety scores, which may result from the nocebo instruction. The difference in state anxiety might have influenced the present findings; however, mean scores of both groups indicate very moderate levels of state anxiety. Lastly, the bar electrode used in the present design might have led to muscle contraction artifacts contaminating SEP findings. Given the very similar stimulation intensities between groups, artifacts might not explain group differences. The problem of potential artifacts could be addressed in futures studies for instance by using ring electrodes (57).

### Conclusion and Outlook

The present study demonstrated an interaction of emotions and reinforced expectations on pain processing. We showed that a placebo manipulation (verbal instruction + placebo conditioning) is able to modulate and even reverse the genuine pain-increasing effect of unpleasant pictures. We assume that the placebo manipulation altered the processing of emotional pictures themselves, such that unpleasant pictures, expected to exert a positive effect on pain, were perceived as less arousing. This interpretation is in line with previous research demonstrating the modulatory influence of threat manipulations on physiological correlates of emotion processing (51, 58). These findings underline the important role of higher order expectations on pain processing and the effectiveness of psychological placebo effects as shown previously (18).

These processes deserve further explorations in future studies, investigating the interaction of placebo/nocebo expectations with other well-established emotional and cognitive factors impacting pain. For instance, it might be worthwhile to investigate whether a nocebo expectation of, e.g., pain exacerbation caused by highly demanding cognitive tasks, actually hampers the pain decrease following manipulations of attention allocation (59, 60). The same might be true for the modulation of pain by emotion regulation strategies such as reappraisal or suppression (61). A placebo vs. nocebo manipulation suggesting high vs. low effectiveness of pain regulation might block or even potentiate its pain-modifying capacities.

## Data Availability

Data is available upon request.

## Ethics Statement

All subjects gave written informed consent in accordance with the Declaration of Helsinki. The protocol was approved by the institutional review board of the medical faculty of the University of Würzburg.

## Author Contributions

PR and MW designed the study. CM collected the data. PR analyzed the data. PR, MW, CM, and PP wrote the manuscript.

## Funding

This work was funded by the Deutsche Forschungsgemeinschaft (DFG, German Research Foundation) to project number 44541416-TRR 58 (B01), and to the Research Group ‘’Emotion and Behavior,’’ FOR 605, Wi2714/3-2. This publication was funded by DFG and the University of Würzburg in the funding programme “Open Access Publishing.”

## Conflict of Interest Statement

The authors declare that the research was conducted in the absence of any commercial or financial relationships that could be construed as a potential conflict of interest.
